# Supplementary material for: Mitotic Catastrophe Induced in HeLa Tumor Cells by Photodynamic Therapy with Methyl-aminolevulinate
Source: Int J Mol Sci. 2019 Mar 11;20(5):1229. doi: 10.3390/ijms20051229 (PMC6429057; doi:10.3390/ijms20051229)
Supplement: Supplementary file 1 [file ijms-20-01229-s001.zip › ijms-449031 Suppl.pdf]

Supplementary

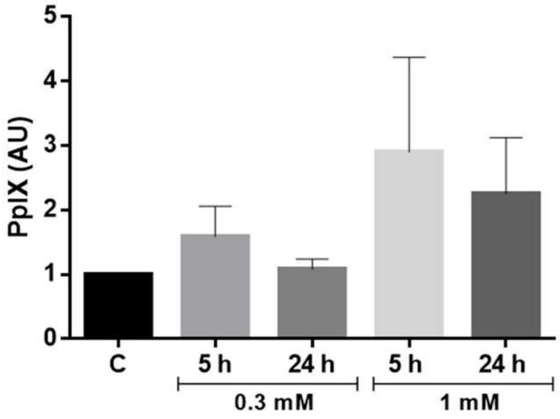

Figure S1. PpIX production in HaCaT cells after 0.3 or 1 mM MAL incubation for 5 or 24 h. Values were relativized to the basal PpIX level in controls (no MAL incubation). Each value corresponds to the mean ± SD obtained from 3 independent experiments.

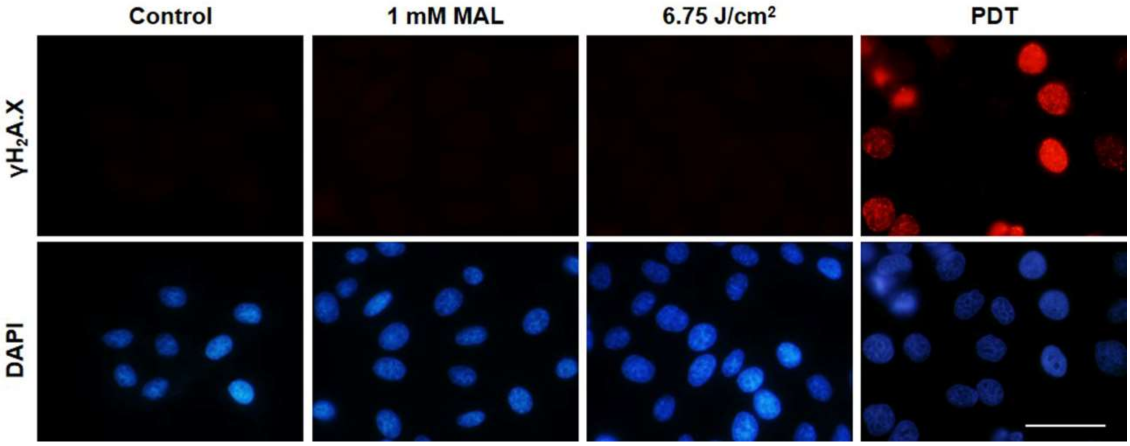

Figure S2. γH2A.X expression determined by IF in untreated cells/controls), in cells exposed to 1 mM MAL (5 h) or to light (6.75 J/cm^2) alone and after PDT. Scale bar: 50 μm.

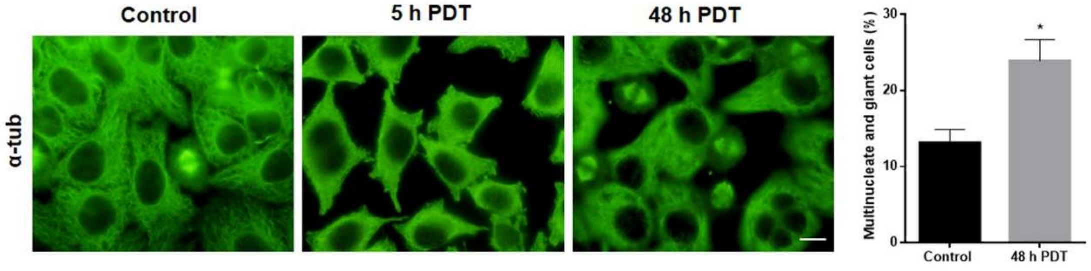

Figure S3. Microtubular alterations 5 and 48 h after PDT determined by IF for α-tubulin. Percentage of multinucleate and giant cells 48 h after PDT. (* $p < 0.05$). Scale bar: 10 μm.

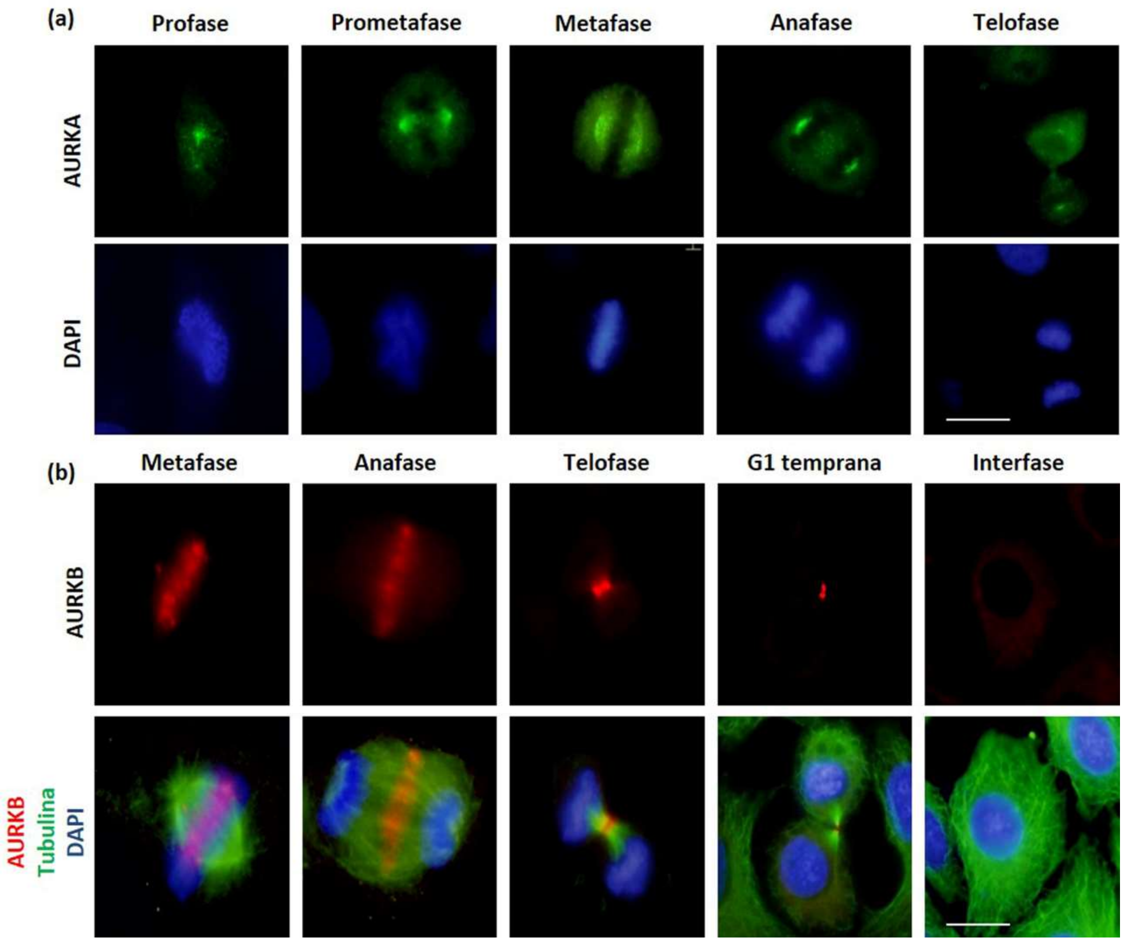

Figure S4. Location of AURKA (**a**) and AURKB (**b**) along cell cycle phases of control HeLa cells. Scale bar: 10 μm.

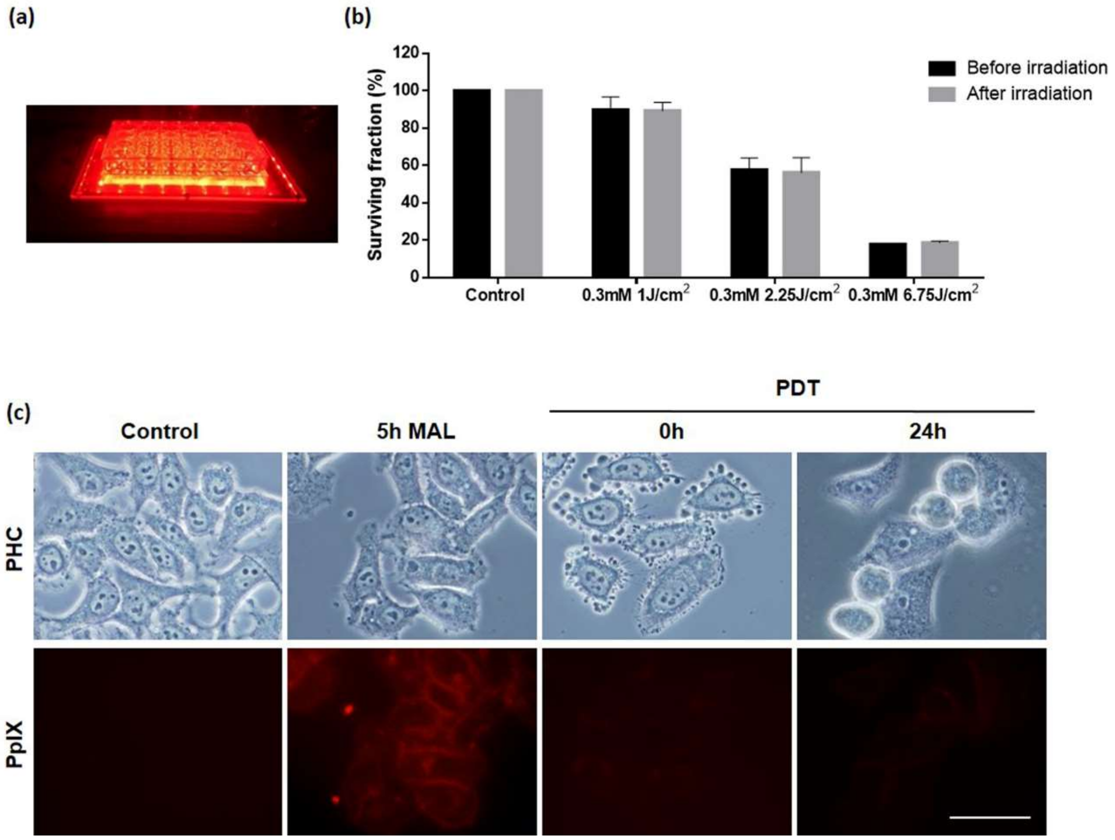

Figure S5. (**a**) Image during irradiation of 24 well plates; irradiation was performed from the bottom of the plates. (**b**) Surviving fraction after MAL-PDT refreshed before/after irradiation. (**c**) Red PpIX fluorescence expression in control cells (without MAL incubation), after 5 h incubation with MAL, and immediately and 24 h after PDT. Scale bar: 50 μm.
